# 20 Years of triple-valve surgery in the UK: demographic and outcome trends

**DOI:** 10.1093/ejcts/ezae268

**Published:** 2024-07-10

**Authors:** Fadi Ibrahim Al-Zubaidi, Nabil Hussein, Harry Smith, Ahmed Al-Adhami, Daniel Sitaranjan, Massimo Caputo, Gianni D Angelini, Amer Harky, Hunaid Ahmed Vohra

**Affiliations:** Department of Cardiac Surgery, Bristol Heart Institute, Bristol, UK; Department of Cardiac Surgery, Castle Hill Hospital, Hull, UK; Department of Cardiac Surgery, Royal Papworth Hospital, Cambridge, UK; Department of Cardiac Surgery, Royal Papworth Hospital, Cambridge, UK; Department of Cardiac Surgery, Royal Papworth Hospital, Cambridge, UK; Department of Cardiac Surgery, Bristol Heart Institute, Bristol, UK; Department of Cardiac Surgery, Bristol Heart Institute, Bristol, UK; Department of Cardiac Surgery, Liverpool Heart & Chest, Liverpool, UK; Department of Cardiac Surgery, Bristol Heart Institute, Bristol, UK

**Keywords:** Mitral valve repair, Mitral valve replacement, Aortic valve replacement, Tricuspid valve repair, Tricuspid valve replacement, Triple valve surgery

## Abstract

**OBJECTIVES:**

To describe evolving demographic trends and early outcomes in patients undergoing triple-valve surgery in the UK between 2000 and 2019.

**METHODS:**

We planned a retrospective analysis of national registry data including patients undergoing triple-valve surgery for all aetiologies of disease. We excluded patients in a critical preoperative state and those with missing admission dates. The study cohort was split into 5 consecutive 4-year cohorts (groups A, B, C, D and E). The primary outcome was in-hospital mortality, and secondary outcomes included prolonged admission, re-exploration for bleeding, postoperative stroke and postoperative dialysis. Binary logistic regression models were used to establish independent predictors of mortality, stroke, postoperative dialysis and re-exploration for bleeding in this high-risk cohort.

**RESULTS:**

We identified 1750 patients undergoing triple-valve surgery in the UK between 2000 and 2019. Triple valve surgery represents 3.1% of all patients in the dataset. Overall mean age of patients was 68.5 ± 12 years, having increased from 63 ±12 years in group A to 69 ± 12 years in group E (*P* < 0.001). Overall in-hospital mortality rate was 9%, dropping from 21% in group A to 7% in group E (*P* < 0.001). Overall rates of re-exploration for bleeding (11%, *P* = 0.308) and postoperative dialysis (11%, *P* = 0.066) remained high across the observed time period. Triple valve replacement, redo sternotomy and poor preoperative left ventricular ejection fraction emerged as strong independent predictors of mortality.

**CONCLUSIONS:**

Triple-valve surgery remains rare in the UK. Early postoperative outcomes for triple valve surgery have improved over time. Redo sternotomy is a significant predictor of mortality. Attempts should be made to repair the mitral and/or tricuspid valves where technically possible.

**Figure:**
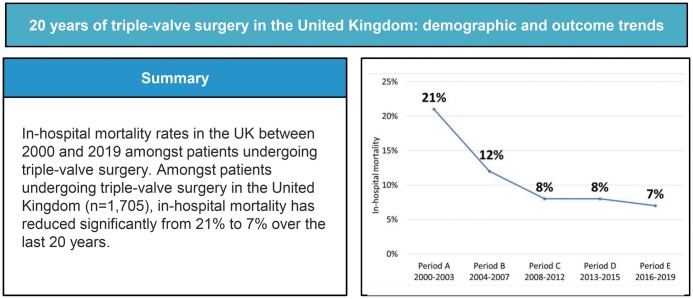

## INTRODUCTION

The UK and many other developed nations are experiencing a significant demographic shift towards a more elderly population [1–3]. In the field of cardiac surgery, this has resulted in a greater proportion of elderly patients presenting for surgery with degenerative valve disease [1, 4]. Patients with severe triple valve disease may undergo triple-valve surgery (TVS), usually involving the repair or replacement of the mitral valve (MV) as well as replacement of the tricuspid valve (TV) and aortic valve (AV).

Degenerative MV disease classically manifests as myxomatous degeneration, characterized by thickening of the mitral leaflets, prolongation or rupture of the chordae tendinae and systolic prolapse of the MV leaflets into the left atrium [5, 6]. This causes progressive regurgitation of blood into the left atrium and pulmonary vasculature during systole. TV regurgitation is most commonly a consequence of prolonged MV regurgitation secondary to raised pulmonary vascular pressures and compensatory right ventricular hypertrophy [7]. This results in right atrial hypertrophy and dilation of the TV annulus. The aetiology of TV regurgitation is therefore described as ‘functional’, as there is no disruption to the structural integrity of the TV apparatus, but rather the leaflets are pulled apart by the dilating annulus leading to impaired coaptation [8]. Degenerative AV disease is most commonly characterized by calcification and stenosis of the AV leaflets, resulting in left ventricular hypertrophy. Patients present with exertional dyspnoea, and in severe cases, this may be associated with angina or syncope.

The literature on TVS largely comprises small, single-centre studies based on data from over a decade ago. The results of these studies demonstrate a high in-hospital mortality rate between 10.8% and 16.1% [9–11]. Given that degenerative valve disease is associated with advancing age, we would expect an increase in patients undergoing TVS. We therefore sought to interrogate the UK National Adult Cardiac Surgery Audit (NACSA) data, maintained by the National Institute for Cardiovascular Outcomes Research (NICOR) [12], to describe and summarize the demographic trends as well as postoperative outcomes in this unique, higher-risk patient population.

## MATERIALS AND METHODS

### Data sources

Our analyses were conducted on the NACSA data, maintained by the NICOR. This national database contains clinical information on demographics, pre- and postoperative clinical information, including mortality, for all major adult cardiac surgery procedures performed in the UK. This national dataset is mandatory and relies on unit-submitted data to NICOR. Maintenance and validation are regularly undertaken by the use of reproducible cleaning and maintenance algorithms, with return to individual centres for local validation [13]. We obtained approval to conduct this study from NICOR and from our institution, the University Hospitals Bristol and Weston National Health Service Foundation Trust Clinical Audit Team, to carry out this research without requiring patient informed consent. The data were received with all identifiable patient information removed; the requirement for patient informed consent was waived and the dataset was adapted for use with statistical analysis software. We received complete data for all patients undergoing MV surgery in the UK, including all patients undergoing concomitant aortic and tricuspid surgery.

### Missingness

The nature of missingness in the dataset was investigated using Little’s test, which returned a significant result (*P* < 0.001), indicating that data were not completely missing at random. In the following variables, we determined that missingness was likely to be related to the true value (percentage missingness stated in brackets): coronary disease (31%), concomitant tricuspid surgery (67%), concomitant AV surgery (58%), concomitant coronary artery bypass graft (29%), concomitant ablation (26%), in-hospital mortality (2%), postoperative stroke (11%), postoperative dialysis (10%) and takeback to theatre (8%). We therefore imputed missing values with ‘0’ in these variables. In the remaining variables, no attempts were made to replace missing values.

### Ethics statement

The register-based cohort study is part of a research approved by the Health Research Authority (HRA) and Health and Care Research Wales and a need for patients’ consent was waived, as all patients in the database were anonymised (HCRW) (IRAS ID: 257758,23.7.2019).

### Study population

This study was conducted utilizing a large dataset of all patients undergoing MV surgery between 2000 and 2019. We selected patients from that dataset undergoing concomitant AV and TV surgery, therefore identifying triple valve surgical patients. We included concomitant coronary artery bypass graft surgery and concomitant ablation for atrial fibrillation (AF). We excluded patients with endocarditis, concomitant major aortic surgery, patients presenting in a critical pre-operative state as well as patients with missing data for procedure date.

### Outcomes

The primary outcome was in-hospital mortality. Secondary outcomes included in-hospital postoperative stroke/transient ischaemic attack, re-exploration for bleeding and post-operative dialysis.

### Statistical analysis

Patients were divided into 5 groups based on date of surgery:

- Period A (2000–2003)
- Period B (2004–2007)
- Period C (2008–2011)
- Period D (2012–2015)
- Period E (2016–2019)

Descriptive statistics were used to compare patient characteristics, intraoperative variables and early postoperative outcomes between these groups. Continuous variables were tested for normality, with normally distributed variables reported as means with standard deviations and non-normally distributed variables reported as median with interquartile ranges. Categorical variables were reported as counts and percentages. For the time-trends analysis, we analysed unadjusted trends for all variables and outcome measures. Trends in categorical variables and continuous variables between the time periods were investigated using Cochran–Mantel–Haenszel tests or simple linear regression, respectively. Simple linear regression was performed on the raw data for each continuous variable. Significance was defined as *P* < 0.05.

We then planned binary logistic regression models to assess the relationship between patient comorbidities and concomitant procedures with all outcome measures. We examined the available variables in the dataset and, based on our clinical judgement, selected the following confounders *a priori*: procedure, sex, age, body mass index, New York Heart Association (NYHA) class, diabetes, hypertension, urgent admission, redo sternotomy, preoperative stroke/transient ischaemic attack, preoperative AF, left ventricular ejection fraction (LVEF), concomitant coronary artery bypass grafting, year of surgery. Adjusted relationships were expressed as odds ratios (OR) with 95% confidence intervals (CI). Complete-case analyses were planned, including cases with complete data after imputing missing values as described above.

## RESULTS

**Figure 1:**
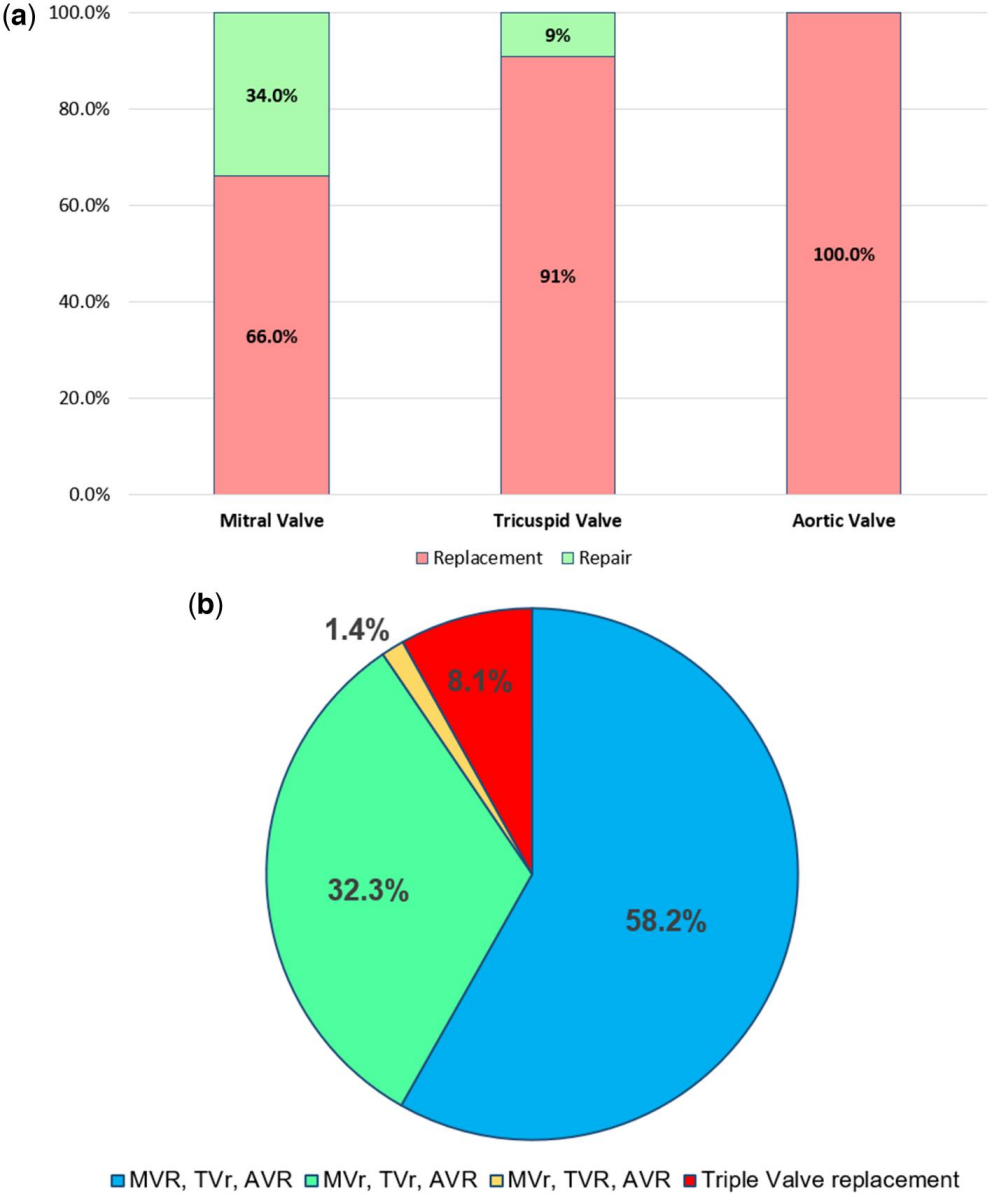
(**a**) Repair rate for mitral, tricuspid and aortic valve procedures. (**b**) Distribution of different procedure types. AVR: aortic valve replacement; MVr: mitral valve repair; MVR: mitral valve replacement; TVr: tricuspid valve repair; TVR: tricuspid valve replacement.

From the overall cohort of 63 808 patients undergoing MV surgery, we identified a total of 2041 triple-valve surgical patients in the UK between 2000 and 2019. After excluding patients presenting in a critical preoperative state (*n* = 265) and those with missing admission date (*n* = 26), we arrived at a final study population of 1750 patients. Patients either received MV and AV replacement with TV repair (*n* = 1019), MV and TV repair with AV replacement (*n* = 565), MV repair with TV and AV replacement (*n* = 24) or triple valve replacement (*n* = 142) (see Fig. 1a and b).

### Volume of triple valve surgery amongst patients undergoing mitral valve surgery

**Figure 2:**
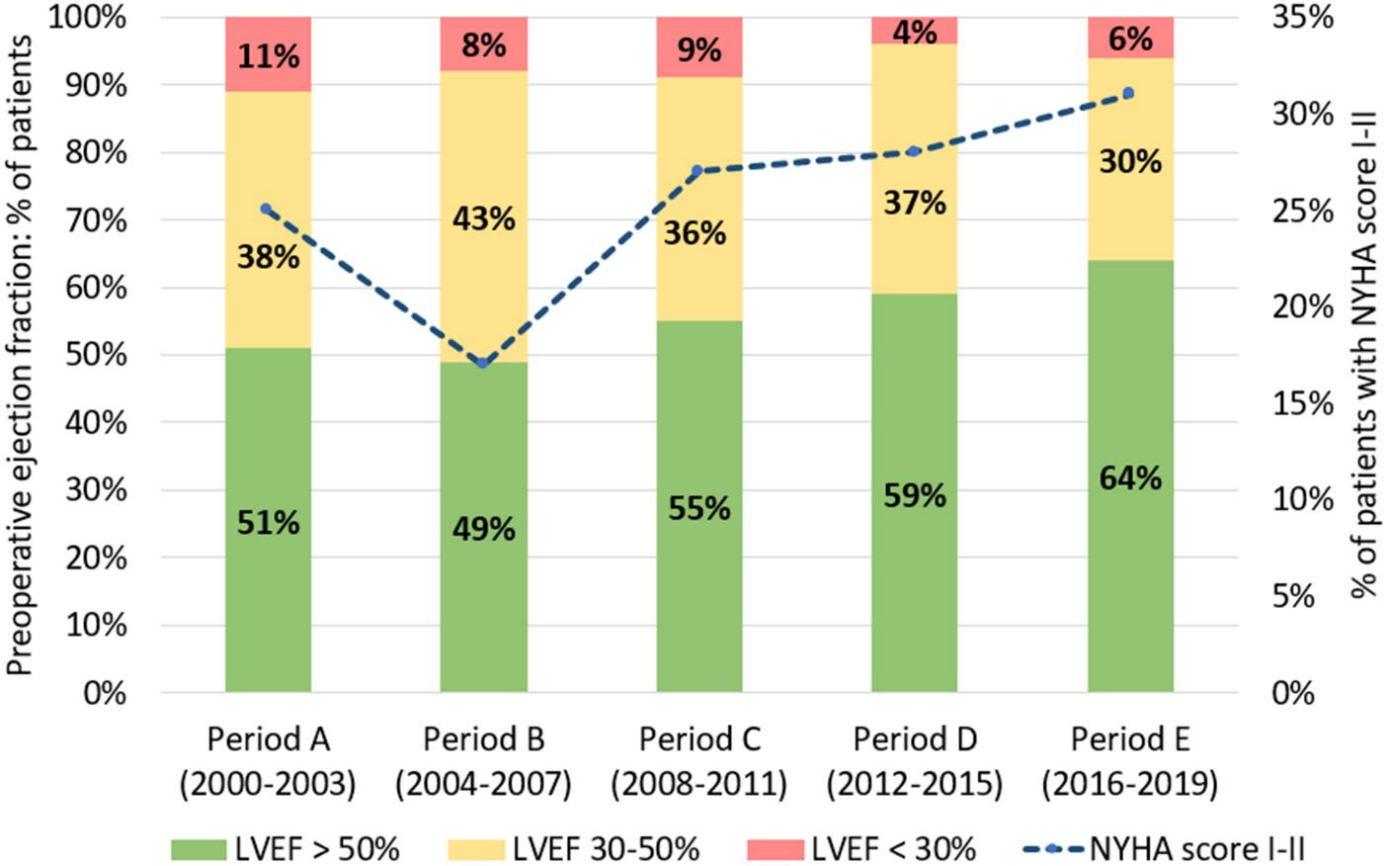
Volume of triple valve surgery and mitral valve surgery. LVEF: left ventricular ejection fraction; NYHA: New York Heart Association.

Rates of TVS amongst patients undergoing MV surgery more than doubled from 1.4% in period A to 3.8% in period C, before plateauing at 3.1% in period E (*P* < 0.001) (see Fig. 2).

### Baseline patient characteristics

**Figure 3:**
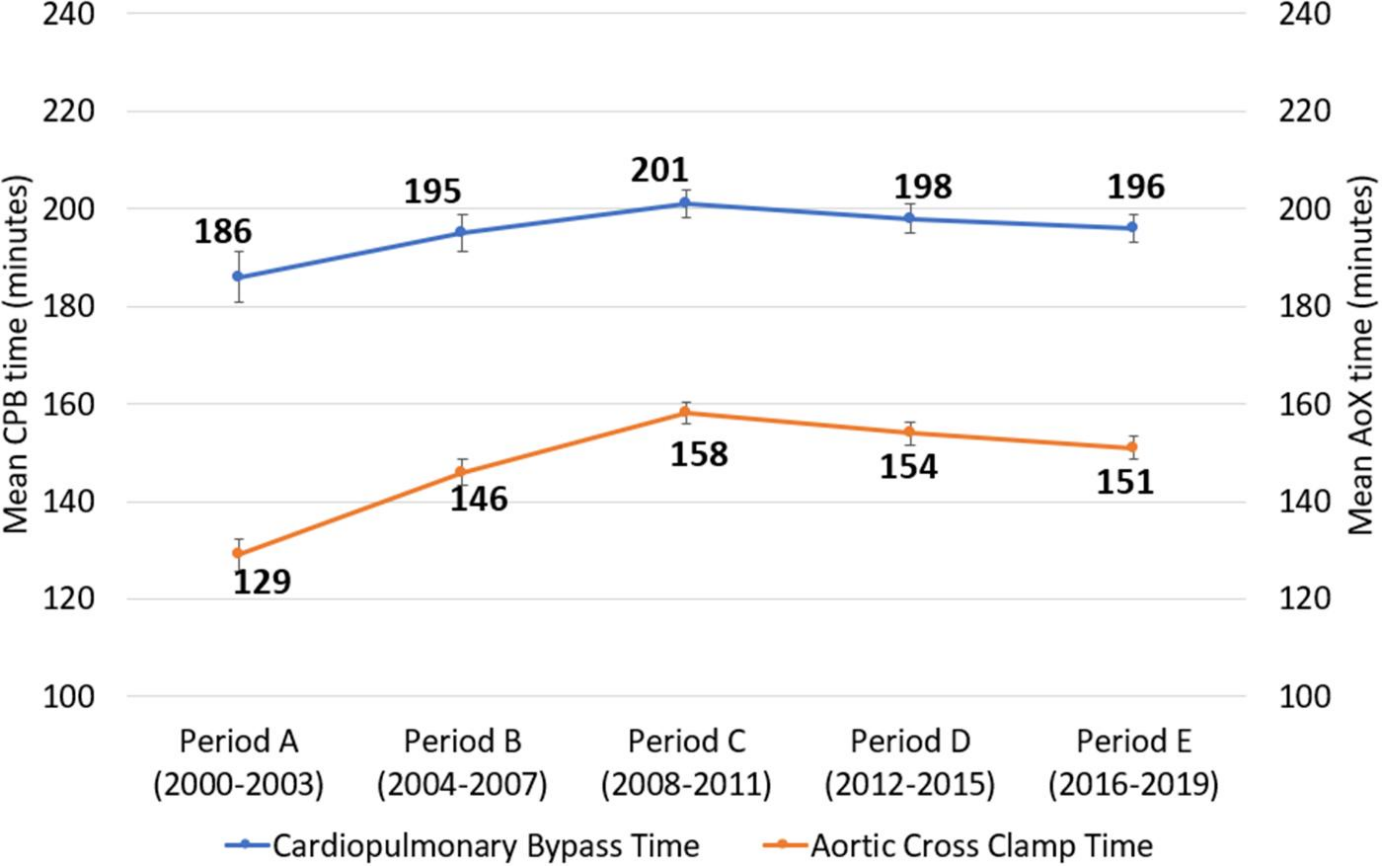
Trends in preoperative left ventricular ejection fraction and low NYHA score. CPB: Cardiopulmonary bypass; NYHA: New York Heart Association.

The mean age in this cohort has risen from 63 ± 12 years in period A to 69 ± 12 years in period E (*P* < 0.001). Mean body mass index has also risen significantly from 24.3 ± 4 kg/m^2^ in period A to 26.9 ± 5 kg/m^2^ in period E (*P* < 0.001). Women make up the majority of this population across the observed time period; however, the sex difference has decreased over time, with women making 59% of TVS patients in period A and 52% by period E (*P* = 0.003). Rates of patients presenting with advanced Canadian Cardiovascular Society angina scores (IV or V) have risen from 6% in period A to 9% in period E (*P* = 0.011); however, rates of patients with advanced New York Heart Association scores (III–IV) dropped from 75% in period A to 69% in period E (*P* = 0.001). Rates of good pre-operative LVEF improved over time, from 51% in period A to 64% in period E (*P* < 0.001) (Fig. 3). Rates of hypertension rose significantly from 28% in period A to 57% in period E (*P* < 0.001). Pre-operative AF decreased from 65% in period A to 54% in period E (*P* = 0.010).

Rates of diabetes, active smoking, previous myocardial infarction, previous stroke or transient ischaemic attack, coronary artery disease and peripheral vascular disease remained stable over time in this cohort (see Table 1).

**Table 1:** Patient demographic trends

Variable	Overall (*n* = 1750)	A: 2000–2003 (*n* = 86)	B: 2004–2007 (*n* = 307)	C: 2008–2011 (*n* = 496)	D: 2012–2015 (*n* = 455)	E: 2016–2019 (*n* = 406)	Trends *P*-value
Age (years)	68.5 (12)	63 (12)	67 (12)	69 (12)	69 (12)	69 (12)	<0.001
BMI (kg/m^2^)	26.3 (5)	24.3 (4)	26.2 (5)	25.9 (5)	26.6 (5)	26.9 (5)	<0.001
Female sex	1009 (58%)	51 (59%)	195 (64%)	295 (60%)	256 (56%)	212 (52%)	0.003
NYHA score							0.001
I–II	457 (26%)	21 (25%)	52 (17%)	134 (27%)	125 (28%)	125 (31%)	
III–IV	1279 (74%)	62 (75%)	248 (83%)	358 (73%)	330 (73%)	281 (69%)	
CCS score							0.011
0	1089 (63%)	46 (56%)	163 (56%)	304 (62%)	295 (65%)	281 (69%)	
I–II	503 (29%)	31 (38%)	106 (36%)	152 (31%)	126 (28%)	88 (22%)	
III–IV	136 (8%)	5 (6%)	22 (8%)	38 (8%)	34 (8%)	37 (9%)	
Diabetes	257 (15%)	8 (10%)	54 (18%)	67 (14%)	65 (14%)	63 (16%)	0.846
Hypertension	940 (54%)	24 (28%)	153 (51%)	268 (55%)	263 (58%)	232 (57%)	<0.001
Active smoker	124 (7%)	9 (11%)	18 (6%)	30 (6%)	35 (8%)	32 (8%)	0.629
Redo sternotomy	324 (19%)	30 (35%)	61 (20%)	88 (18%)	79 (17%)	66 (16%)	0.003
Preoperative COPD	311 (18%)	12 (14%)	51 (17%)	95 (19%)	92 (20%)	61 (15%)	0.960
Previous stroke/TIA	204 (12%)	7 (9%)	36 (12%)	53 (11%)	58 (13%)	50 (12%)	0.448
Vascular disease	136 (8%)	4 (5%)	25 (8%)	48 (10%)	39 (9%)	20 (5%)	0.226
Preoperative AF	1000 (59%)	54 (65%)	189 (64%)	279 (60%)	258 (60%)	220 (54%)	0.010
Coronary disease	407 (23%)	10 (12%)	80 (26%)	123 (25%)	108 (24%)	86 (21%)	0.874
EF range							<0.001
>50%	982 (57%)	41 (51%)	146 (49%)	272 (55%)	266 (59%)	257 (64%)	
30–50%	621 (36%)	30 (38%)	128 (43%)	175 (36%)	167 (37%)	121 (30%)	
<30%	129 (7%)	9 (11%)	27 (9%)	44 (9%)	22 (5%)	27 (7%)	

AF: atrial fibrillation; BMI: body mass index; CCS: Canadian Cardiovascular Society Score; COPD: chronic obstructive pulmonary disease; EF: ejection fraction; NYHA: New York Heart Association; TIA: transient ischaemic attack.

### Intra-operative variables

Rates of urgent admission remained stable over time at around 29% (*P* = 0.317). Rates of MV repair have risen significantly in this population, from 12% in period A to 38% in period E (*P* < 0.001). Mean cardiopulmonary bypass times remained stable over time around 197 ± 62 min (*P* = 0.323). Mean aortic cross-clamp times rose significantly from 129 ± 30 min in period A to 152 ± 46 min in period E (*P* < 0.001). All AV procedures were AV replacements. Rates of TV repair in this cohort have risen significantly over time, from 12% in period A to 16% in period E (*P* < 0.001). Rates of concomitant coronary artery bypass grafting remained stable around 23% over time (*P* = 0.118). Amongst patients with AF, rates of concomitant AF ablation rose from 0% in period A to 8% in period C and 20% in period E (*P* < 0.001).

### Postoperative outcomes

**Figure 4:**
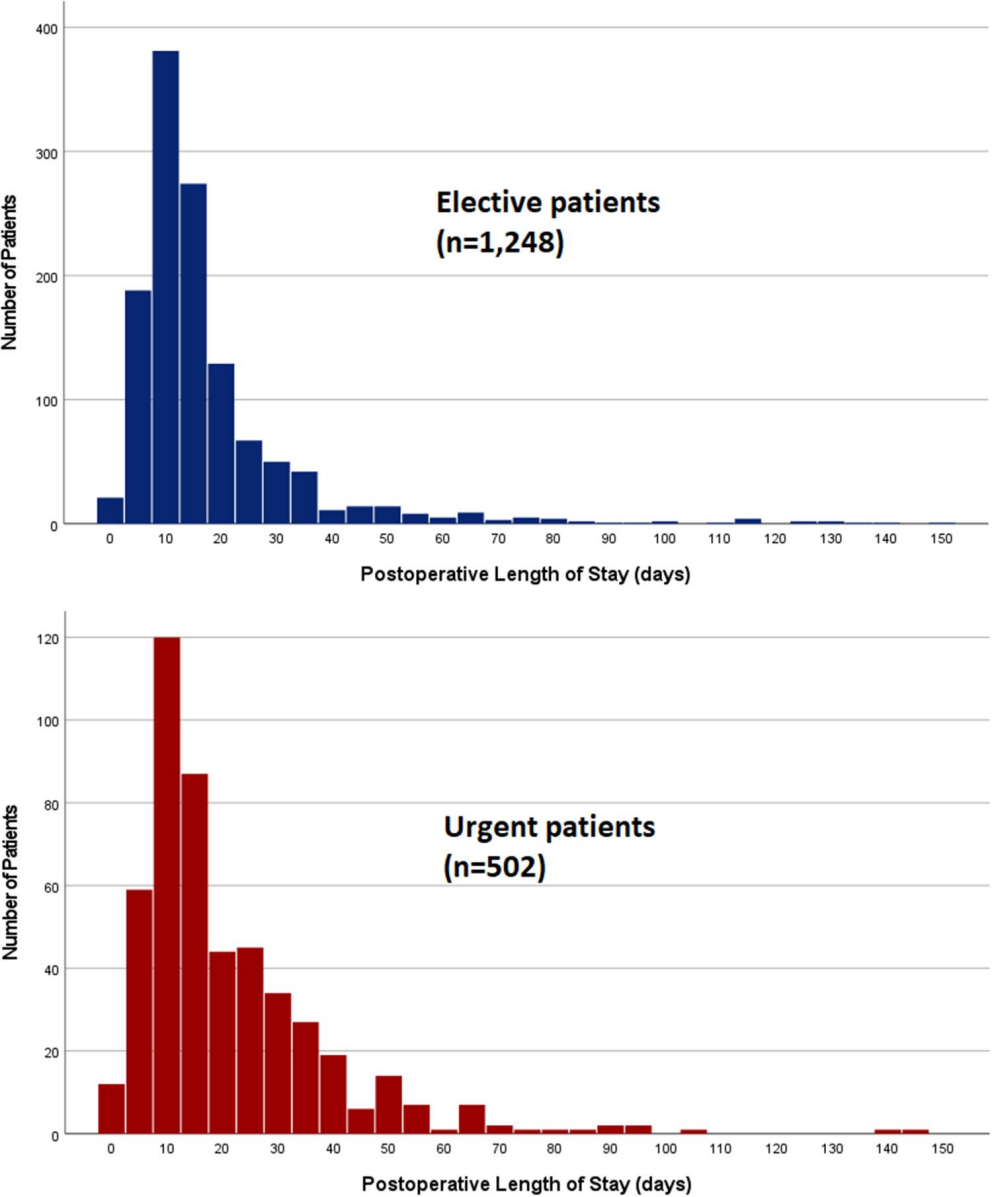
Admission length histogram (days).

In-hospital mortality rates have improved significantly, dropping from 21% in period A to 8% in period C and 7% in period E (*P* < 0.001). There were no significant changes in rates of postoperative stroke (*P* = 0.284) or re-exploration for bleeding (*P* = 0.308). Amongst elective patients, mean length of stay dropped significantly from 18 days in period A to 16 days in period E (*P* = 0.044). By contrast, there was no significant change in length of stay amongst patients undergoing urgent surgery (*P* = 0.593) (Fig. 4). Rates of deep sternal wound infection rose from 0% in period A to 2% in period E (*P* = 0.006). Postoperative dialysis rates remained stable around 11% (*P* = 0.066).

### Regression analysis

#### Procedure type

A total of 1586 (91%) patients were included in the complete-case regression analyses for in-hospital mortality, postoperative stroke, re-exploration for bleeding and postoperative dialysis. Procedure type was independently associated with mortality and stroke. Combinations of MV replacement, TV repair and AV replacement (OR 1.74, 95% CI 1.10–2.75, *P* = 0.017) as well as patients undergoing triple valve replacement (OR 4.02, 95% CI 2.01–8.07, *P* < 0.001) were independently associated with in-hospital mortality. MV replacement, TV repair and AV replacement were the only independent predictors for postoperative stroke (OR 2.60, 95% CI 1.10–6.14, *P* = 0.029). There was no independent association between procedure type and postoperative dialysis; 164 (9%) patients with incomplete data were excluded from the regression analysis.

#### Patient factors

Additional independent predictors of in-hospital mortality were advancing age (OR 1.03, 95% CI 1.01–1.06, *P* < 0.001), NYHA class III–IV (OR 1.74, 95% CI 1.05-2.90, *P* = 0.033), urgent admission (OR 1.60, 95% CI 1.09–2.37, *P* = 0.017), redo sternotomy (OR 3.15, 95% CI 2.12–4.67, *P* < 0.001), moderate LVEF (OR 1.71, 95% CI 1.15–2.53, *P* = 0.008) and poor LVEF (OR 2.25, 95% CI 1.21–4.17, *P* = 0.010). Additional independent predictors of postoperative stroke were advancing age (OR 1.05, 95% CI 1.01–1.09, *P* = 0.022) and diabetes (OR 3.06, 95% CI 1.39–6.74, *P* = 0.005). Later year of surgery was a consistent protective factor against postoperative stroke.

Male sex (OR 1.72, 95% CI 1.22–2.40, *P* = 0.002) was the only independent predictor of re-exploration for bleeding. Later year of surgery was not a protective factor against re-exploration. Diabetes, urgent admission and redo sternotomy were all found to be independent predictors of postoperative dialysis.

#### Time period

Odds ratios for mortality decreased significantly with each time period, signalling a significant overall improvement in the management of patients undergoing TVS. Advancing time period was also a protective factor against stroke in each time period (see Table 4).

**Table 4:** Multivariable associations with primary and secondary outcomes

Variable	Outcome			
	Mortality	Stroke/TIA	Re-exploration	Dialysis
Procedure combination				
MVr, TVr, AVR	Reference	Reference	Reference	Reference
MVR, TVr, AVR	**1.74 (1.10–2.75) *P* = 0.017**	**2.60 (1.10–6.14) *P* = 0.029**	0.73 (0.51–1.05) *P* = 0.088	0.85 (0.59–1.25) *P* = 0.411
MVr, TVR, AVR	3.18 (0.79–12.86) *P* = 0.104	3.68 (0.39–34.61) *P* = 0.255	0.80 (0.18–3.59) *P* = 0.771	0.96 (0.21–4.48) *P* = 0.958
MVR, TVR, AVR	**4.02 (2.01–8.07) *P* < 0.001**	1.62 (0.31–8.29) *P* = 0.565	1.20 (0.65–2.20) *P* = 0.565	1.27 (0.66–2.46) *P* = 0.468
Male sex	1.43 (0.97–2.10) *P* = 0.072	1.62 (0.80–3.30) *P* = 0.181	**1.72 (1.22–2.40) *P* = 0.002**	1.41 (1.00–2.00) *P* = 0.052
Age (years)	**1.03 (1.01**–**1.06) *P* = 0.001**	**1.05 (1.01**–**1.09) *P* = 0.022**	1.00 (0.98–1.01) *P* = 0.920	1.01 (1.00–1.03) *P* = 0.085
BMI (kg/m^2^)	0.98 (0.94–1.02) *P* = 0.379	0.99 (0.92–1.06) *P* = 0.800	1.00 (0.96–1.03) *P* = 0.859	1.03 (1.00–1.07) *P* = 0.064
NYHA class III–IV	**1.74 (1.05**–**2.90) *P* = 0.033**	1.37 (0.57–3.26) *P* = 0.481	1.06 (0.73–1.54) *P* = 0.773	1.41 (0.91–2.16) *P* = 0.120
Diabetes	1.32 (0.81–2.15) *P* = 0.262	**3.06 (1.39–6.74) *P* = 0.005**	1.15 (0.74–1.79) *P* = 0.531	**2.29 (1.55–3.37) *P* < 0.001**
Hypertension	0.97 (0.67–1.42) *P* = 0.888	0.57 (0.28–1.16) *P* = 0.123	1.06 (0.76–1.47) *P* = 0.744	1.03 (0.73–1.45) *P* = 0.869
Urgent admission	**1.60 (1.09–2.37) *P* = 0.017**	1.18 (0.56–2.46) *P* = 0.665	1.32 (0.93–1.88) *P* = 0.118	**2.07 (1.46–2.93) *P* < 0.001**
Redo sternotomy	**3.15 (2.12–4.67) *P* < 0.001**	0.90 (0.37–2.18) *P* = 0.818	1.26 (0.85–1.88) *P* = 0.256	**1.73 (1.17–2.55) *P* = 0.006**
Preoperative stroke/TIA	1.20 (0.72–2.01) *P* = 0.486	0.69 (0.23–2.08) *P* = 0.515	0.93 (0.57–1.52) *P* = 0.776	1.40 (0.89–2.20) *P* = 0.142
Preoperative AF	1.33 (0.90–1.95) *P* = 0.153	1.96 (0.98–3.93) *P* = 0.057	0.82 (0.58–1.16) *P* = 0.262	0.88 (0.62–1.26) *P* = 0.491
LVEF				
>50%	Reference	Reference	Reference	Reference
30–50%	**1.71 (1.15–2.53) *P* = 0.008**	1.09 (0.51–2.32) *P* = 0.823	1.02 (0.72–1.44) *P* = 0.919	1.03 (0.73–1.47) *P* = 0.854
<30%	**2.25 (1.21–4.17) *P* = 0.010**	2.70 (0.99–7.40) *P* = 0.053	1.27 (0.72–2.24) *P* = 0.406	1.24 (0.70–2.22) *P* = 0.464
Concomitant CABG	1.49 (0.98–2.26) *P* = 0.059	0.96 (0.43–2.14) *P* = 0.926	0.94 (0.64–1.39) *P* = 0.762	0.95 (0.65–1.41) *P* = 0.810
Year of surgery				
2000–2003	Reference	Reference	Reference	Reference
2004–2007	0.54 (0.26–1.16) *P* = 0.115	**0.20 (0.06**–**0.70) *P* = 0.011**	0.60 (0.27–1.34) *P* = 0.214	1.55 (0.60–4.00) *P* = 0.363
2008–2011	**0.37 (0.17–0.78) *P* = 0.009**	**0.08 (0.02–0.33) *P* < 0.001**	0.87 (0.41–1.86) *P* = 0.726	1.35 (0.53–3.44) *P* = 0.528
2012–2015	**0.32 (0.15–0.69) *P* = 0.004**	**0.32 (0.10–0.97) *P* = 0.044**	0.72 (0.33–1.55) *P* = 0.399	0.99 (0.39–2.57) *P* = 0.991
2016–2019	**0.24 (0.11–0.54) *P* < 0.001**	**0.19 (0.06–0.64) *P* = 0.007**	0.49 (0.22–1.09) *P* = 0.081	0.76 (0.29–1.99) *P* = 0.575

Bold indicates statistical significance.

AF: atrial fibrillation; AVR: aortic valve replacement; BMI: body mass index; CABG: coronary artery bypass grafting; LVEF: left ventricular ejection fraction; MVr: mitral valve repair; MVR: mitral valve replacement; NYHA: New York Heart Association; TIA: transient ischaemic attack; TVr: tricuspid valve repair; TVR: tricuspid valve replacement.

## DISCUSSION

In this study, we seek to describe the shifting demographic and early outcome trends of patients undergoing TVS in the UK utilizing the NACSA data. We have demonstrated that in-hospital mortality rates have improved significantly, dropping from 21% in 2000–2003 to 7% in 2016–2019. By contrast, rates of re-exploration for bleeding and postoperative dialysis have remained high, both at 11%. There has been an overall rise in the volume of patients undergoing TVS. We have identified a number of clinically important predictors of mortality as well as all secondary outcomes.

The proportion of MV patients undergoing TVS in this study rose slightly over time from 1.4% to 3.1% (see Fig. 2). The proportion of cardiac surgical patients undergoing TVS in the UK is therefore likely comparable to figures cited by other nations, which usually cite a rate of 1–2% [9, 14–17]. Given the ageing population [2] and overall rise in patients with multiple valve disease [18], one may have expected to see a steeper rise in patients undergoing TVS. The consistently low numbers across different regions likely reflects a number of important factors such as high reported operative risk [9, 14–16], technical difficulty and patient reluctance.

An alternative explanation for the low numbers of patients undergoing TVS is the improved medical management of patients with heart failure reducing the need for patients to undergo surgery. A range of therapeutic advances in recent years such as the use of sodium–glucose co-transporter 2 inhibitors [19, 20], previously an antidiabetic medication, have contributed significantly to improvements in patient symptom profiles and long-term prognosis [21]. This appears to be reflected in our analysis, which shows that a greater proportion of patients are presenting for TVS with lower NYHA scores as well as better LVEF (see Fig. 3), likely indicating an overall improvement in medical optimization of patients with multi-valve disease in the UK. These findings are even more striking given the mean patient age rising by 6 years over the same time period (see Table 1) to a mean of 69 years ± 12, higher than many other papers looking at TVS [22–25].

The UK in-hospital mortality rate has seen a remarkable drop from 21% in 2000–2003 to 7% in 2016–2019 (see Table 3), a low figure when compared against those quoted in other studies [9, 14, 16]. This is despite significant rises in mean age, obesity and the rates of hypertension, preoperative AF and redo sternotomy in this cohort (see Table 1). Improvements in pre-operative optimization, intraoperative technique and postoperative care for patients may all explain this change. Our multivariable analysis demonstrated time to be an independent protective factor against mortality, supporting the view that there has been a general improvement in the clinical management of these patients. Another explanation to consider is the shifting aetiologies of disease over this time period.

**Table 3:** Postoperative outcome trends

Variable	Overall (*n* = 1750)	A: 2000–2003 (*n* = 86)	B: 2004–2007 (*n* = 307)	C: 2008–2011 (*n* = 496)	D: 2012–2015 (*n* = 455)	E: 2016–2019 (*n* = 406)	Trends *P*-value
In-hospital mortality, *n* (%)	160 (9)	18 (21)	38 (12)	40 (8)	36 (8)	28 (7)	<0.001
Stroke/TIA, *n* (%)	45 (3)	6 (7)	8 (3)	8 (2)	14 (3)	9 (2)	0.284
Re-exploration, *n* (%)	197 (11)	12 (14)	30 (10)	64 (13)	55 (12)	36 (9)	0.308
Median length of stay (days), median (IQR)	13 (9–22)	13 (8–20)	13 (8–20)	15 (10–24)	13 (8–19)	13 (8–20)	0.160
Elective mean length of stay	13 (9–22)	12.5 (8–18)	12 (8–18)	14 (10–22)	13 (8–20)	13 (8–20)	0.234
Urgent mean length of stay	15 (10–30)	17 (7–33)	15 (10–30)	17 (10–33)	14 (10–22)	14 (10–22)	0.048
DSWI, *n* (%)	15 (1)	0 (0)	0 (0)	2 (0)	7 (2)	6 (2)	0.006
Dialysis, *n* (%)	183 (11)	7 (8)	41 (13)	58 (12)	44 (10)	33 (8)	0.066

DSWI: deep sternal wound infection; TIA: transient ischaemic attack.

Triple valve replacement emerged as the strongest independent predictor of mortality (see Table 4). One explanation for this is that it is a surrogate marker for disease severity, as patients with the most extensive valve disease would be selected for triple valve replacement. It may also be a surrogate marker for patient comorbidity status. Furthermore, less experienced surgeons may opt to replace all valve more frequently, and so triple valve replacement may be a surrogate marker for relatively less surgical experience.

Whilst improving mortality rates and preoperative optimization of patients are positive, it is important to emphasise that our findings show patients undergoing TVS to have significantly longer operations, more complicated and longer admissions when compared to more routine procedures [4, 6]. As expected, they have significantly long bypass and cross-clamp times (see Table 2). One in 10 patients are re-explored postoperatively for bleeding, and 1 in 10 will require postoperative dialysis (see Table 3). Diabetes emerged as an independent predictor of postoperative dialysis, an important finding given the sharp rise in diabetes in the general population [26].

**Table 2:** Operative variable trends

Variable	Overall (*n* = 1750)	A: 2000–2003 (*n* = 86)	B: 2004–2007 (*n* = 307)	C: 2008–2011 (*n* = 496)	D: 2012–2015 (*n* = 455)	E: 2016–2019 (*n* = 406)	Trends *P*-value
Urgent admission, *n* (%)	502 (29)	23 (27)	103 (34)	131 (26)	137 (30)	108 (27)	0.317
MV procedure, *n* (%)							<0.001
Repair	589 (34)	10 (12)	77 (25)	195 (39)	154 (34)	153 (38)	
Replacement	1161 (66)	76 (88)	230 (75)	301 (61)	301 (66)	254 (62)	
TV procedure *n* (%)							<0.001
Repair	1584 (91)	76 (88)	296 (96)	469 (95)	402 (88)	341 (84)	
Replacement	166 (9)	10 (12)	11 (4)	27 (5)	53 (12)	65 (16)	
CPB time (min), mean (SD)	197 (62)	186 (46)	195 (64)	200 (61)	198 (66)	196 (58)	<0.001
AoX time (min), mean (SD)	152 (46)	129 (30)	146 (44)	158 (50)	154 (50)	152 (46)	<0.001
Ablation *n* (%)	101 (10)	0 (0)	0 (0)	21 (8)	37 (14)	43 (20)	

AoX: aortic cross-clamp; CPB: cardiopulmonary bypass; MV: mitral valve; TV: tricuspid valve.

Overall, we have demonstrated TVS remains a rare procedure in the UK. Whilst outcomes have improved over time, this remains a high-risk cohort. Individual risk factors such as diabetes and previous cardiac surgery should be taken into account when counselling patients and weighing up risks. The strengths of our analyses include the large sample from a national audit, making our findings reasonably generalizable to the UK population. In most variables, there was a low degree of missingness.

### Limitations

Our conclusions need to be interpreted in light of limitations associated with a nonrandomized, retrospective study design. Whilst the NACSA data are regularly maintained and validated, it is possible that some data may have been reported incorrectly. Further granularity into the specific valvular lesions, surgical techniques used and whether a repair was attempted before replacement would have allowed for a more rigorous analysis. Unfortunately, we did not have access to data on patient ethnicity, the mode of surgical incision or rates of postoperative permanent pacemaker implantation. These are important trends to capture going forward, particularly as the adoption of minimally invasive techniques continues to rise. Finally, we did not have data on long-term outcomes (e.g. mortality, stroke, hospital readmission, etc.) because the NACSA dataset is not linked with hospital episode or mortality data.

## CONCLUSIONS

1. In-hospital mortality has reduced significantly from 21% to 7%.
2. Attempts should be made to repair the mitral and/or TVs where technically feasible.
3. TVS remains rare in the UK.

## Data Availability

The data underlying this article were provided by the National Institute for Cardiovascular Outcomes Research (NICOR) by permission. Data will be shared on request to the corresponding author with permission of NICOR.

## ABBREVIATIONS

AF: Atrial fibrillation
AV: Aortic valve
CI: Confidence intervals
HCRW: Health and Care Research Wales
HRA: Health research authority
MV: Mitral valve
NACSA: National Adult Cardiac Surgery Audit
NICOR: National Institute for Cardiovascular Outcomes Research
NYHA: New York Heart Association
OR: Odds ratios
TV: Tricuspid valve
TVS: Triple-valve surgery
